# Cotrimoxazole Prophylaxis Specifically Selects for Cotrimoxazole Resistance in *Streptococcus mutans* and *Streptococcus sobrinus* with Varied Polymorphisms in the Target Genes *folA* and *folP*

**DOI:** 10.1155/2012/916129

**Published:** 2012-01-24

**Authors:** Buwembo William, Charles Mugisha Rwenyonyi, Göte Swedberg, Fred Kironde

**Affiliations:** ^1^Department of Anatomy, College of Health Sciences, Makerere University, P.O. Box 7072, Kampala, Uganda; ^2^Department of Dentistry, Makerere University, Kampala, Uganda; ^3^Department of Medical Biochemistry and Microbiology, Uppsala University, 75123 Uppsala, Sweden; ^4^Department of Biochemistry, Makerere University, Kampala, Uganda

## Abstract

The selection of antibiotic resistance by cotrimoxazole prophylaxis was evaluated, and we characterized the mechanism of cotrimoxazole resistance in *Streptococcus mutans* and *Streptococcus sobrinus*. *In vitro* susceptibility to six antibiotics was evaluated on 64 mutans streptococci group (MSG) isolates from a cotrimoxazole prophylaxis group and compared to 84 MSG isolates from a nonprophylaxis group. The *folA* and *folP* genes were sequenced and compared with reference sequences at NCBI. Only resistance to cotrimoxazole was significantly higher in the prophylaxis group (54.7% versus 15.5%, OR = 6.59, 95% CI: 2.89–15.3, *P* < 0.05). Resistance to amoxicillin, ceftriaxone, chloramphenicol, erythromycin, and tetracycline was 1.4%, 25.5%, 6.2%, 6.5%, and 29.6% of the isolates, respectively. Considerable polymorphisms were found in the *folP* gene in *S. mutans*, but this could not be linked to sulfonamide drug resistance. No variation was seen in *folP* or *folA* genes of *S. sobrinus*. Genetic transfer of folate pathway genes seems unlikely in these isolates.

## 1. Introduction

The mutans streptococci group (MSG) belong to the viridans group streptococci (VGS) and form an important part of the normal oral flora [1, 2]. Although the VGS are generally nonpathogenic, they have attracted attention due to their ability to act as reservoirs for antibiotic resistance genes and to transfer these troublesome traits to classically pathogenic bacteria including *Streptococcus pneumoniae* and *Streptococcus pyogenes *[3]. While a number of recent studies [4, 5] have investigated the susceptibility patterns of the VGS to various antimicrobial agents, only a few studies [6] have reported antimicrobial sensitivity of VGS from sub-Saharan Africa even though antibiotic abuse is widespread in the region [7]. More specifically, it has been observed that cotrimoxazole (SXT) prophylaxis selects for resistance in infectious streptococci [8, 9]. However, information on the effects of SXT prophylaxis on commensal bacteria which are not the drug targets is still scarce, particularly in sub-Saharan Africa bearing a heavy burden of human immunodeficiency virus infection/acquired immunodeficiency syndrome (HIV/AIDS) and where a large proportion of the patients routinely take SXT as prophylaxis against *Pneumocystis jiroveci *pneumonia.

The MSG includes seven species of which *Streptococcus mutans* and *Streptococcus sobrinus* infect human populations [10]. As the principal causative agents of dental caries (tooth decay) and related odontogenic pyogenic infections, the MSG is commonly referred to as cariogenic bacteria [11]. They are also implicated in causing extra-oral infections, particularly subacute bacterial endocarditis in patients with defective or prosthetic valves [12, 13]. Assessment of antibiotic susceptibility and characterisation of the mechanisms of the resistance of the MSG is increasingly important not only because these bacteria are the leading cause of dental and serious extra-oral infections but also because they can carry and transfer antibiotic resistance genes to typically pathogenic microbes [14]. Additionally, to our knowledge the mechanism of *Streptococcus mutans* and *Streptococcus sobrinus* resistance to cotrimoxazole has not been characterised.

Here, we have evaluated the selection of antibiotic resistance by SXT prophylaxis and characterised the mechanism of *Streptococcus mutans* and *Streptococcus sobrinus* resistance to SXT. The bacteria were isolated from saliva of patients attending Mulago Hospital Dental Clinic and The AIDS Support Organization (TASO) clinic in Kampala, Uganda.

## 2. Methods

### 2.1. Study Areas

This study was conducted in TASO Clinic and Mulago Hospital Dental Clinic located in Kampala, Uganda. TASO is a nongovernmental HIV/AIDS care clinic that attends to about 100 patients daily who regularly take SXT as prophylaxis. Mulago Hospital is the national referral hospital. It has a dental clinic which daily offers treatment to about 50 outpatients whose HIV sero-status is undetermined and is not taking SXT as prophylaxis.

### 2.2. Study Design and Subjects

This was a cross-sectional study of patients (*n* = 204) from TASO Clinic who were routinely taking SXT as prophylaxis (prophylaxis group) and another group of patients (*n* = 171) from Mulago Dental clinic that were not taking SXT as prophylaxis (nonprophylaxis group). To increase chances of isolating MSG, the patients were selected on condition that they had at least one tooth with dental caries confirmed by clinical examination. Demographic data and information on use of antibiotics in the previous three months were obtained from patients' interview and medical records.

### 2.3. Ethical Considerations

Permission to carry out this study was obtained from Makerere University School of Medicine Research and Ethics Committee and the Uganda National Council of Science and Technology. Written consent was sought and obtained from each study subject consistent with the Helsinki Declaration relating to the conduct of research on human subjects [15].

### 2.4. Microbiological Procedures and Antibiotic Resistance

All the selected patients (*n* = 375) donated saliva specimens for the study. At least 1 h following the patient's meal, 2 mL of whole saliva was collected in a sterile falcon tube (Sarstedt, Austria), and within 1 h of being collected, the specimen were transported to the Department of Biochemistry, Makerere University College of Health Sciences for analysis. Each specimen was diluted 1 : 100 in physiological saline (Chemtrec, USA) and 100 *μ*L of the diluted sample was cultured on plates containing modified HLR-S medium [16] for selective isolation of MSG. The plates were incubated at 37°C for 48 h in an atmosphere of 5% carbon dioxide. One presumptive colony of MSG was picked and streaked on chocolate agar plates (BioMérieux, France), supplemented with 5% sheep blood and polyvitex (BioMérieux, France). The colonies from chocolate agar were identified by morphology, gram staining, haemolysis on blood agar medium (BioMérieux, France), catalase test, and susceptibility to optochin [2]. Subsequent differentiation of the bacteria was done by biochemical tests [2, 17] and confirmed by polymerase chain reaction (PCR) genotyping of the glycosyl transferase (*gtf*) gene [10]. After confirmation of the bacterial species by PCR, susceptibility to amoxicillin and cotrimoxazole was evaluated using *E*-test (AB Biodisk, Sweden) whereas susceptibility to ceftriaxone, erythromycin, tetracycline, and chloramphenicol was assessed by the Kirby Bauer disc diffusion method (BioMérieux, France) [18]. The break points criteria for streptococcal species other than *Streptococcus pneumoniae* established by the CLSI [18] were used for all the antibiotics studied except for cotrimoxazole in which the break points established by the Swedish Reference Group for Antibiotics [19] were adopted.

### 2.5. Deoxyribonucleic Acid (DNA) Extraction

Isolates of MSG were incubated at 37°C for 12 h on chocolate agar. Bacteria from one colony were resuspended in Trypticase Soy broth (BioMérieux, France) and incubated at 37°C for 24 h in an atmosphere of 5% carbon dioxide. Bacterial DNA from the cultured streptococci was then extracted using a modified protocol of the Wizard Genomic DNA Purification Kit (Promega, USA).

### 2.6. Polymerase Chain Reaction for Species Identification

Sequences of the primers used in PCR confirmation of bacterial species were as previously described [10]. In this procedure, a DNA fragment (517 bp) of the *gtfB *gene of *Streptococcus mutans *or a longer fragment (712 bp) of the *gtfI *gene of *Streptococcus sobrinus* was amplified. Briefly, each PCR mixture of 20 *μ*L contained DreamTaq green buffer (Fermentas, Lithuania), 100 *μ*M each of dATP, dTTP, dGTP, and dCTP, 0.5 *μ*M oligonucleotide primers (both GTFB-F, -R and GTFI-F, -R), 1.25 U of DreamTaq polymerase (Fermentas, Lithuania), and 0.5 to 1 nanogram of template DNA.

The PCR conditions comprised 30 cycles of denaturation at 95°C for 30 s, annealing at 59°C for 30 s, and extension at 72°C for 1 min. The amplified products were separated by electrophoresis in 0.8% agarose gels (Sigma, Belgium) and stained with ethidium bromide (BDH, UK).

### 2.7. Statistical Analysis

The data were analyzed using the Statistical Package for Social Sciences Inc. (version 12.0 for Windows, Chicago, IL, USA). Frequency distribution was used to describe the material. The dependent variable (SXT prophylaxis use) was categorized as 1 = yes and 2 = no. Binary logistic regression analyses were used to estimate the magnitude of the risk of bacteria in developing drug resistance in the prophylaxis and nonprophylaxis groups. The 95% confidence intervals were generated around all odds ratios (OR) to determine the significance of predictor variables. A *P* value of <0.05 was considered statistically significant.

### 2.8. Sequence Analysis

DNA from a number of isolates was sequenced using the BigDye Terminator labelled cycle sequencing kit (Applied Biosystems) and an ABI prism 310 Genetic Analyzer (Applied Biosystems) in Uppsala University Sweden. Primers used for DNA amplification and sequencing reactions are listed in Table 1. The results of the *folA* and *folP* gene sequence analysis were compared with database sequences of *S. mutans* UA 159 [20] and NN2025 [21] using the BLAST programme at NCBI. Additionally, the *folP* gene of isolate 797 was compared to a number of organisms using the multiple sequence alignment program clustalW at the European Bioinformatics Institute database.

### 2.9. Cloning of the *folP* Gene from Strain 797

Isolate 797 with most point mutations was further characterised. The *folP* gene of strain 797 was amplified using primers MdhpsSph and MdhpsBam (Table 1) where recognition sequences for the restriction enzymes *Sph*I and *Bam*HI were engineered into the forward and reverse amplification primers, respectively. The resulting PCR product was digested with these two enzymes together with the plasmid vector pUC19. After ligation of PCR product and vector using the Rapid Ligation Kit (Fermentas, Lithuania), the resulting plasmid was used to transform competent cells of *E. coli* DH5*α*. After verification of the plasmid construction, it was introduced into the *E. coli* knock-out strain C600Δ*folP *[22].

### 2.10. Cloning and Sequencing of the *folP* Gene from *Streptococcus sobrinus*

No full genome sequence of *S. sobrinus* is available, but the sequence of the closely related species *S. downei* was recently added to the databases. Thus, primers were designed to match the *S. downei* sequence as shown in Table 1. PCR was performed with an annealing temperature of 45°C; otherwise, the PCR conditions were as described previously. Sequencing reactions and analysis were as described previously.

### 2.11. Selection of Trimethoprim Resistant Variants of *Streptococcus mutans* Strain 797

When cultivated in the lab, *S. mutans* strain 797 showed stable resistance to sulfamethoxazole but susceptibility to trimethoprim (tp). The strain was cultivated in Isosensitest Broth (ISB) (Oxoid, UK) with a low concentration (2 *μ*g/mL) of tp, where growth was very weak. From this culture, the bacteria were inoculated into tubes containing ISB with 5 *μ*g/mL of tp and from this culture subsequently reinoculated into ISB with 10 *μ*g/mL of tp. From the latter two cultures, bacteria were streaked onto Isosensitest Agar (ISA) plates (Oxoid,UK) containing 10 *μ*g/mL of tp. Bacteria from both tubes grew on the selective plates and were called 797 (5) and 797 (10), respectively.

### 2.12. Competition Experiments

Bacteria strains 797(10) and 797 were inoculated in the 2 mL of Brain Heart Infusion (BHI) (BioMérieux, France) in the following proportions: Tube 1, 100 *μ*L 797(10); Tube 2, 10 *μ*L 797(10) + 90 *μ*L 797; Tube 3, 50 *μ*L 797(10) + 50 *μ*L 797; Tube 4, 90 *μ*L 797(10) + 10 *μ*L 797; and Tube 5, 100 *μ*L 797. Additionally, 100 *μ*L of 797(10) and 797 diluted 10 times was spread on ISA plates (Oxoid,UK) in order to get individual colonies, which was counted to identify the initial proportions.

The five cultures were then incubated overnight at 37°C in a 5% CO_2_ atmosphere. At a 6 hourly interval: 0, 6, 12, 18, and 24 hours, 10 *μ*L of bacteria was removed from each test tube and diluted 100 times in BHI broth (BioMérieux, France). One hundred *μ*L of the dilutions was then spread on ISA plates (Oxoid, UK) and incubated overnight at 37 degrees in a 5% CO_2_ atmosphere. The following day, individual colonies were picked and inoculated on ISA plates (Oxoid,UK) with and without trimethoprim 10 *μ*g/mL. This experiment was done three times and the averages of the proportion of resistant to susceptible colonies grown on the ISA plates were counted and plotted against time.

## 3. Results

### 3.1. Antibiotic Resistance Patterns in the Prophylaxis Group (*n* = 204)

Based on culture and *gtf* genotyping, 31.4% (*n* = 64) of the patients' specimens had MSG strains. Thirty six (56%) of the strains were *S. mutans* and 28 (44%) were *S. sobrinus*. There were no statistically significant differences in the resistance pattern of the various antibiotics between *S. sobrinus* and *S. mutans *(*P* > 0.05); thus the data were pooled. Cotrimoxazole showed the highest resistance (54.7%), while amoxicillin had the lowest resistance (1.6%, Table 2). The risk of developing resistance to SXT in the prophylaxis group was 6.59 times higher than that in the nonprophylaxis group (*P* < 0.05, Table 2). For other antibiotics, there were no significant differences in resistance rates between the two study groups. The risk of developing multidrug resistance (MDR), that is, resistance to more than 2 drugs in the prophylaxis group, was 1.42 times higher than that in the nonprophylaxis group. However, the difference was not statistically significant (95% CI: 0.55–3.71, Table 3). Based on the individual components of SXT (sulfamethoxazole and trimethoprim), most (95%) of the isolates in the prophylaxis group showed resistance to sulfamethoxazole, while 61% of resistance was towards trimethoprim.

### 3.2. Antibiotic Resistance Patterns in the Nonprophylaxis Group (*n* = 171)

In the nonprophylaxis group, 51% (*n* = 87) of the patients had positive growth of MSG bacteria. According to *gtf* genotyping, 37 (43%) of the strains were *Streptococcus mutans* and 47 (54%) were *Streptococcus sobrinus* while 3 (3.4%) of the specimens contained both species. The most prevalent resistance was associated with tetracycline (35.7%), while the least resistance was found with erythromycin (3.6%, Table 2). Resistance to cotrimoxazole in the nonprophylaxis group was 15.5%. Coresistance of the isolates to different antibiotics is shown in Table 3. Overall, MDR was observed in 14% of the nonprophylaxis specimens.

### 3.3. Determination of *folA* Sequences in Susceptible and Resistant Isolates

**Figure 1:**
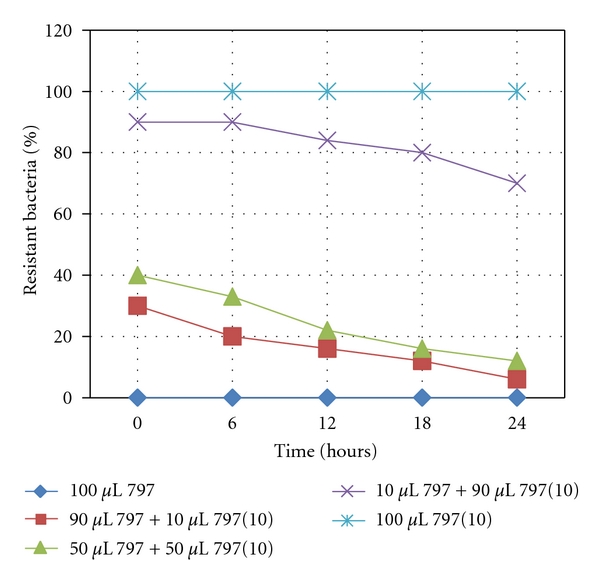
Plot of resistant bacteria after competition between susceptible 797 and resistant 797 (10).

Sequence analysis of the *folA* gene from *S. mutans* as compared to the database sequences UA159 and NN2025 revealed no point mutations irrespective of the susceptibility status of the strain. One isolate, 797, was chosen for more detailed analysis. It was originally isolated as a trimethoprim (tp) resistant strain but lost the resistance upon subculturing in the lab. Further application of trimethoprim selective pressure on the strain in the lab led to its rapid development of resistance to trimethoprim. When the two lab isolates 797 (5) and 797 (10) were derived and selected on 5 *μ*g/mL and 10 *μ*g/mL of trimethoprim, respectively, the full nucleotide sequence for the *thyA*, a gene coding for a putative transporter, and *folA* genes were determined including the upstream sequence of *thyA*, but no single nucleotide change was detected in this region. Based on the lab-derived tp resistant strains in competition experiments with the original 797, the tp susceptible isolate 797 was found to be more fit than the tp resistant 797(10) (Figure 1). The tp susceptible isolate outgrew the resistant strain over a 24-hour period even when added in a minority proportion.

### 3.4. Determination of *folP* Sequences

**Figure 2:**
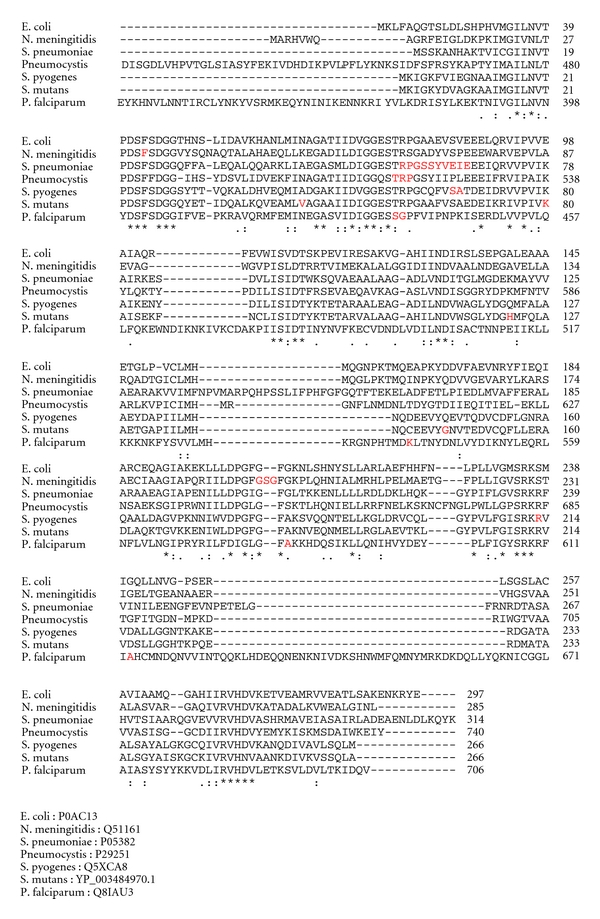
Comparison of the mutations in 797 compared to NN2025 showing that all differ quite substantially from positions where you find resistance determining mutations (in red) in other organisms.

**Figure 3:**
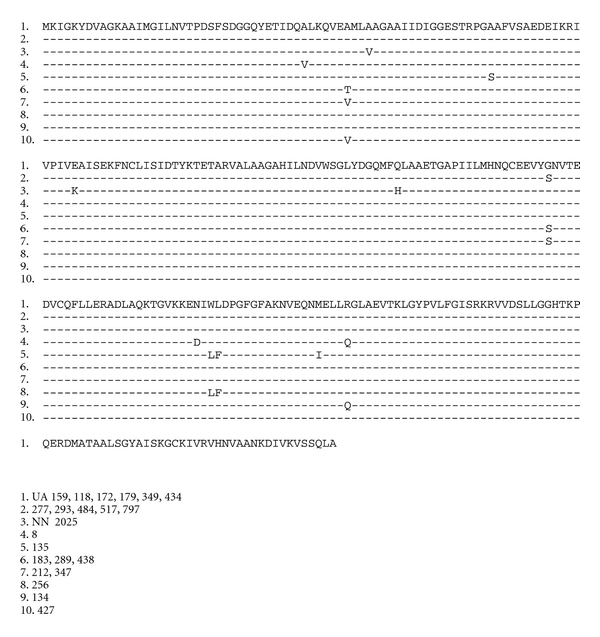
Variations in *S. mutans *DHPS amino acid sequences.

Sequence analysis of the *folP* gene for 797 revealed four point mutations in positions at A46 V, E 80 K, Q122 H, and S146G when compared to the database sequence of strain NN 2025. However, in comparison with the other published sequence from UA 159, only position S146G differed in 797. Further comparison of DHPS from isolate 797 with DHPS from other sulfonamide resistant organisms (Figure 2) showed that the differences occur outside of previously characterised mutation determining positions. The *folP* gene was much more polymorphic than the *folA* gene. Sequence determinations from a number of other isolates showed ten different patterns of changed amino acids (Figure 3). When the *folP* gene from 797 was cloned in the pUC19 vector, it was shown to express DHPS in *E. coli* by transformation of the knock-out strain C600Δ*folP*. The 797 strain readily grew on 0.1 mM sulfamethoxazole, but the cloned *folP* gene only allowed growth on 0.02 mM sulfamethoxazole.

**Figure 4:**
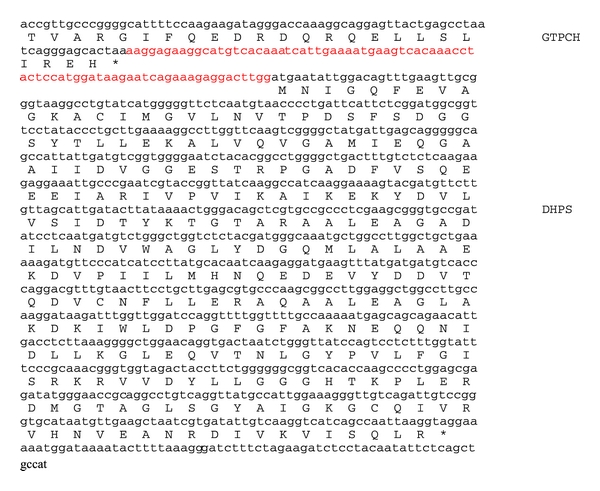
Full *S. sobrinus folP* gene with translation. Red section is missing in *S. downei*.

**Figure 5:**
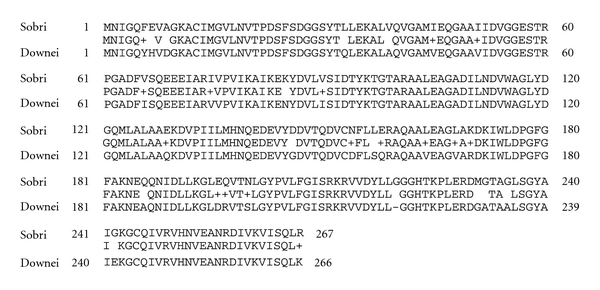
Comparison of DHPS amino acid sequences between *Streptococcus sobrinus* (Sobri) and *Streptococcus downei* (Downei).

No genomic sequence of *S sobrinus* was available and it was initially difficult to get the full *folP* sequence of the isolates, which was finally resolved by designing primers flanking the *folP* gene of *S. downei* with a sequence similar to that of *S sobrinus*. The full *folP* sequence is presented in Figure 4. Variants of isolate 7(0) showing different response to sulfamethoxazole had exactly the same sequence for *folP* and the upstream region. The sequence intervening the genes *folE* (coding for GTP cyclohydrolase) and *folP* is almost 100 bp longer in *S. sobrinus* than in *S. downei *(Figures 4 and 5). The intervening sequence shows no similarity to any sequence in databases and seems thus to be unique to *S. sobrinus *(Figure 5). The *folP* and *folA* gene sequences of *S. sobrinus* together with the *folP* sequences of different variants of *S. mutans* prepared in this study have been submitted to the European Nucleotide Archive with accession numbers HE 599533 to HE 599539.

## 4. Discussion

Viridans group streptococci resistant to antibiotics have increasingly been reported over the past decade [4, 5], while studies on antibiotic resistance of mutans streptococcus group are few [23, 24]. In the present study, cariogenic mutans streptococci (*Streptococcus mutans and Streptococcus sobrinus*) were isolated from patients attending dental and HIV/AIDS care clinics in Kampala, Uganda, where studies on drug resistance in the commensal flora are scarce, but with a reported increased use of antibiotics [7]. The most striking result is the very distinct selection of SXT resistant bacteria in the prophylaxis group, while resistances to other antibiotics were not significantly different between the two groups.

Recently, Wilén et al. [25] reported cotrimoxazole resistance in viridans streptococci from the throat flora and found a 100% frequency of resistance. However, isolates of *Streptococcus mutans* and *Streptococcus sobrinus* were not included in that previous study [25]. In the present study, the low level of resistance (15.5%) found in samples from the nonprophylaxis group might be explained by the different SXT selective pressure in the different environments and that the cariogenic bacteria produce biofilms [26], a protective mechanism that renders the bacteria to survive antibiotic exposure. However, the significantly higher level of resistance found in the prophylaxis group (54.7%) shows that selective forces act on the oral and throat flora. The differences in resistance level may instead be explained better by differences in the way the throat commensal flora develop resistance to SXT [25]. There may also be other limits in the genetic transfer among the oral compared to the throat flora.

When the nonmutans viridans streptococci resistance mechanism to SXT was analysed, it was easily identified as due to mutational changes in the target enzymes dihydropteroate synthase (DHPS) and dihydrofolate reductase (DHFR) as well as genetic transfer between different streptococcal species [25]. However, findings from sequencing of *folP* and *folA* genes from resistant MSG in the present study (Figures 2–5) do not suggest mutational changes in the DHFR enzyme, nor any genetic transfer of resistance determinants from the other viridians group streptococci to *S. mutans* or *S. sobrinus. *The lack of changes in the *folA* gene suggests a different mechanism of resistance to trimethoprim, probably due to gene amplification as has been noted in *S. agalatiae *[27]. On the other hand, the *folP *gene of *Streptococcus mutans* 797 showed four point mutations in comparison with NN2025 (Figure 2) and one in comparison with UA159. Comparison of the positions of these mutations with resistance determining mutations in other organisms (Figure 2) shows that they mainly occur in less conserved parts of the protein and differ quite substantially in positions compared to previously described resistance conferring mutations [22, 25, 28–30]. Further sequencing of other *S. mutans* isolates showed much more polymorphism (Figure 3) and it is evident that the *S. mutans* DHPS can vary quite substantially without any obvious relation in resistance to sulfamethoxazole. The lack of evidence for horizontal transfer of resistance genes from related streptococci is quite striking. During isolation of the cariogenic bacteria, it was very common to find other commensal streptococcal species, for example, *S. sanguinis, S. parasanguinis, *and *S. salivarius* in the same isolate. In a few instances we amplified *folA* and *folP* genes from these other commensal species and found the expected resistance conferring mutations described in the previous study [25]. While the resistance conferring variants of *folA* and *folP* are freely disseminated among most oral streptococcal strains, there seems to be some barrier that limits transfer of resistance to the cariogenic bacteria.

*Streptococcus sobrinus* is quite different from *S. mutans* in terms of DNA sequence for the genes we studied, but also in this case the sulfa-trimethoprim resistance seems not to be linked to sequence differences in *folA* and *folP*. The sequences of these genes show high similarity between *S. sobrinus* and *S. downei *(Figure 5), and in few cases we detected *S. downei* sequences in the samples. The striking difference between the two bacteria is the intergenic region between *folE* and *folP *(Figure 4). This may need to be explored further regarding its relation to expression of *folP* and hence to resistance. However, during cultivation, we obtained *S. sobrinus* strains with varying degrees of sulfa and tp susceptibilities, but no changes were noted in this region.

Despite the protective effect of cotrimoxazole prophylaxis in HIV/AIDS patients from opportunistic infections and malaria [9], the present study found the drug to select for resistance among the mutans streptococci similar to what has been reported in other commensals like *Escherichia coli* [8]. Looking at other individual resistances rates, there are only a few studies that specifically describe *Streptococcus mutans or S. sobrinus*. A study from India [22] showed resistance levels of 20–30% for tetracycline, amoxicillin, and erythromycin. In the present study, resistance of mutans streptococci to tetracycline was 35.7%, which is obviously a worrisome finding because of the modest cost and easy availability of the drug in Uganda.

Resistance to amoxicillin, ceftriaxone, and chloramphenicol was found in 1.2%, 32.1%, and 6%, respectively, of the isolates (Table 2). These resistance levels are similar or higher than those reported from India [22]. Our observed resistance may be attributed to selection of resistant organisms in the study area similar to the finding in India [22], given the prevailing high rate of antibiotic use in the two countries [7].

Coresistance (resistance to more than one antibiotic) or the event where microbes acquire resistance to multiple antibiotics in tandem is extremely troublesome and has previously been noted [22]. Although we found a slightly higher prevalence of multidrug resistance in the prophylaxis as compared to the nonprophylaxis group (Table 3), the difference was not statistically significant. This could partly be explained not only by the fact that most of the antibiotics in the present study are not frequently used in the HIV/AIDS patients but also by the difference between the mechanisms of action of these antibiotics and cotrimoxazole.

These findings call for improved surveillance of antibiotic resistance during prophylaxis. Updated information on antibiotic resistance such as reported in the present study helps to inform antibiotic drug policy makers to design strategies for effective prophylaxis against and treatment of bacterial infections.

## 5. Conclusions

In the present study, it has been found that Cotrimoxazole prophylaxis selects for cognate antibiotic resistance in oral bacteria. There was a high rate of resistance of *Streptococcus mutans* and *Streptococcus sobrinus* to tetracycline and ceftriaxone in the studied Ugandan patients independent of SXT prophylaxis. Genetic transfer in the folate pathway genes does not seem to be the mechanism of resistance acquisition.

## Figures and Tables

**Table 1:** Primers used.

DHFRS-F	TTTCTTAATTTCTGATAGAATG
DHFRS-R1	GCCGTAAACACCCCAAAAAT
DHFRM-F1	TGGTTGGGTAAAATGGGAAC
DHFRM-R	GCATATCTTAAGCCAATC
Operon 11 Reverse	AGAGCATTCAGCAAAAGTCC
Operon 24 Forward	TGTCTGTCATCTTCTGCCC
Operon 24 Reverse	CCTATCACAACCCTTCGTCC
MdhpsSph	GATCGATCGCATGCACATCATAACTAGGGAGCAAGC
MdhpsBam	GATGGATCGGATCCAAAATAATCTTATCCATAACACCCTCA
Dhpssrevnew	GCGGGAAATACCAAAGAGGAC
Gtchsfw	GCCTTGGAAGAAGCCTTAGAC
Gtpchfw2	ACTTTGATTAAGGAGCACTAG
Gtpchfw3	TATGTGTATGACTATGCGAGG
Downei dhps rev1	AAAAGTATTTTATCCATATTC
Downei dhps rev2	CCCTTTAAAAGTATTTTATCCAT

**Table 2:** Comparison of the different antibiotic susceptibilities of the nonprophylaxis and prophylaxis groups*.

Antibiotic		STX prophylaxis use		OR	95% CI
		No *n* (%)	Yes *n* (%)		
Cotrimoxazole	S	71 (84.5)	29 (45.3)	6.59	2.89–15.37
	R	13 (15.5)	35 (54.7)		

Amoxicillin	S	61 (72.6)	41 (64.1)	1.49	0.02–118.67
	R	1 (1.2)	1 (1.6)		
	I	22 (26.2)	22 (34.4)		

Tetracycline	S	38 (45.2)	38 (59.4)	0.50	0.22–1.15
	R	30 (35.7)	15 (23.4)		
	I	16 (19.0)	11 (17.2)		

Erythromycin	S	74 (88.1)	49 (76.6)	3.02	0.63–19.37
	R	3 (3.6)	6 (9.4)		
	I	7 (8.3)	9 (14.1)		

Chloramphenicol	S	72 (85.7)	54 (84.4)	1.07	0.2–5.21
	R	5(6)	4 (6.3)		
	I	7 (8.3)	6 (9.4)		

Ceftriaxone	S	46 (54.8)	50 (78.1)	0.41	0.17–0.96
	R	27 (32.1)	12 (18.8)		
	I	11 (13.1)	2 (3.1)		

S: susceptible; R: resistant; *Intermediate resistance was not used in calculating OR.

**Table 3:** The frequency distribution of coresistance in the nonprophylaxis and the prophylaxis groups.

Number of antibiotics to which isolates were resistant	Nonprophylaxis group *n* (%)	Prophylaxis group *n* (%)
0	32 (37.2)	14 (21.9)
1	23 (26.7)	20 (31.3)
2	19 (22.1)	18 (28.1)
More than 2	12 (14.0)	12 (18.7)

Total	84 (100)	64 (100)
